# Thermodynamics Analysis of a Reaction-Diffusion Matrix Multiplication Computing Unit under the Linear Non-Equilibrium Regime

**DOI:** 10.1021/acs.jpclett.5c00834

**Published:** 2025-06-13

**Authors:** Giuseppe S. Basile, Stefan Angerbauer, Giuseppe Grasso, Werner Haselmayr, Nunzio Tuccitto

**Affiliations:** † Department of Chemical Sciences, 9298 University of Catania, Catania, Viale Andrea Doria 6, 95125, Italia; ‡ Scuola Superiore di Catania, Via Valdisavoia 9, Catania 95123, Italia; ¶ Institute for Communication Engineering and RF-Systems, 27266 Johannes Kepler University Linz, Linz 4040, Austria

## Abstract

Implementations of matrix multiplication via diffusion and reactions, thus eliminating the need for electronics, have been proposed as a stepping stone to realize molecular nano-neural networks (M3N). This work examines whether such ”matrix multiplication units” can function spontaneously, i.e., without continuous external energy input. We employ the theory of local non-equilibrium thermodynamics in the linear regime, modeling the system through coupled reaction-diffusion equations and deriving the resulting entropy production. Numerical simulations on a 2D computational mesh confirm that correct matrix multiplication and strictly increasing entropy can be attained under two key conditions: negligible cross-diffusion among distinct species and sufficiently sharp membranes to prevent back diffusion. When these constraints are met, the system concentrations naturally converge to the desired results, suggesting that autonomous chemical computing can be realized if the design parameters align with thermodynamic requirements.

## Graphical abstract

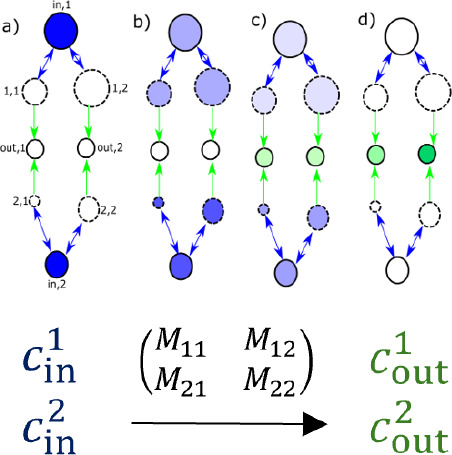

The Internet of Bio-Nano Things (IoBNT) has emerged as a central research focus for communication engineers, chemists, and information theorists over the past two decades.^1–4^ Researchers have invested considerable effort into developing biologically compatible devices capable of performing computations and completing specific tasks on micro- and nanoscopic scales. The long-term goal is to supplement or even replace electronic devices as control entities within the human body. On the edge of this philosophy, there are many possible realizations of nonconventional computing hardware, ranging from chemical reservoirs^5^ to biological computing and memristive arrays.^6^ All these approaches exploit physical phenomena to encode inputs, outputs, and the dynamical evolution of systems, thus enabling, in principle, the physical realization of autonomous computing architectures such as echo state networks.^7^

One promising direction for building computing units that do not rely on electronics involves harnessing nonelectromagnetic physical systems to mimic the signal processing typical of analog or digital computation. This is accomplished by encoding inputs and outputs into the physical evolution of specific intensive or extensive quantities.

Within this framework, numerous solutions for different tasks have been proposed, such as molecular nanoneural networks (M3N), capable of performing simple classification tasks much like traditional neural networks.^8^ M3N, in their original formulation by Angerbauer et al., are based on phenomenological mass transport laws and reaction kinetics. The key advantage of these systems is their ability to operate entirely without electronic components, relying instead on diffusion and reactions to convert inputs in outputs. A crucial stepping stone toward building a functioning M3N, or any neural network, is defining a form of matrix multiplication capable of transforming an input vector into an output vector. While this is relatively straightforward to achieve ”in silico” with common mathematical definitions, translating the problem into a physical system where an input vector is represented by initial concentrations presents a greater challenge. Angerbauer et al. have explored various geometries and implementations in both theoretical and experimental studies, addressing these complexities on both a mathematical level and with macroscopic experimental systems.

The original works on the matrix computing unit focused on the mathematical background, ensuring that the unit effectively fulfills its task, and on stochastic simulations. As a result, they did not emphasize the role of energy dissipation or the thermodynamic assumptions underlying possible realizations of the matrix structure.

As for what concerns the present Letter, we will thus try to answer the question: is it possible for the matrix multiplication unit in question to function spontaneously without a constant supply of external energy?

In other words: what are the physical requirements for a matrix computing unit to work spontaneously without the aid of an external operator during its computing cycle? To do so, we will initially remark how the matrix multiplication is designed from a physical point of view, referring the reader to previous works for details and derivations of the most important relations but stating clearly our assumptions. After this brief presentation, we will introduce a series of thermodynamics arguments based on the theory of Local Non-Equilibrium Thermodynamics in the Linear Regime,^9,10^ exploiting the concept of entropy production in irreversible systems to address the time evolution of concentrations for each of the chemical species involved.

The matrix multiplication unit discussed in this work was originally introduced in^11^ and a detailed analysis was carried out in ref 12. A schematic illustration of it is depicted in Figure 1. Concretely, this structure realizes a 2 × 2 matrix multiplication. Circles indicate compartements, i.e., physical volumes inside which chemicals are distributed homogeneously. Chemicals are indicated by Caslon upper case letters and can move between compartments in the direction indicated by the arrows. Mass transport occurs through the channels connecting the compartments by means of diffusion. These channels are indicated by the lines connecting the circles. The first substance A is present only in the inlets (uppermost row) and intermediate (second row) compartments. Its initial concentration $C_{\mathrm{in},i}^{A,\mathrm{init}}$ encodes the *i*-th input into the computing structure. In the example above, *i* ∈{1,2} as there are two inputs. The final concentration of substance B in the *j*-th outlet, i.e., $C_{\mathrm{out},j}^{B,\mathrm{fin}}$ encodes the outcome of the computation. In the above example *j* ∈{1,2} as there are two outlets.

**Figure 1:**
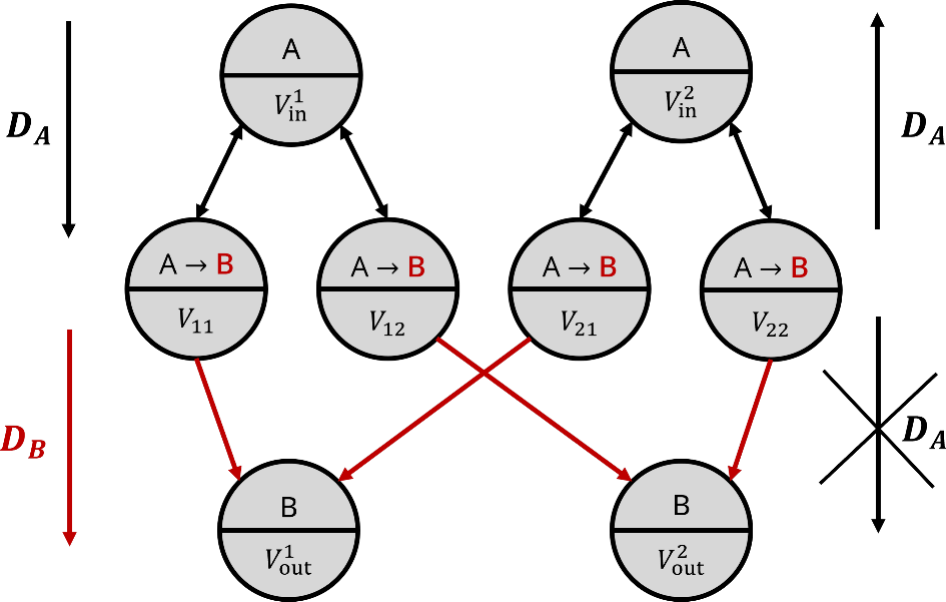
Original version of the computing unit topology, based on irreversible reactions and assuming positively defined matrix entries (thus requiring only one type of reaction, as explained in ref 12). A molecules diffuse freely from inlets to intermediates, B molecules selectively flow from intermediates to outlets.

The system works by combining diffusion through the channels with reactions of type A→B . The reader should note that in this version of the computing unit all reactions are unidirectional, while in the thermodynamics treatment we will shift to reactions of type A⇋B for physical consistency.

In the following, we will provide an intuitive interpretation for the working principle of the computing structure in the form of a video on paper (Figure 2). For a detailed mathematical treatment, we refer the reader to Angerbauer et al.^12^

**Figure 2:**
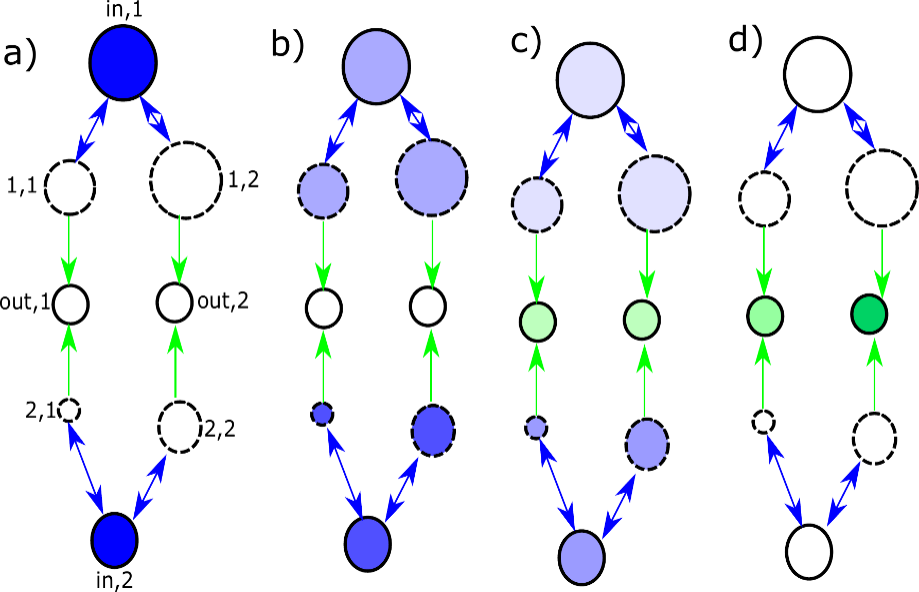
Video on paper shows how the matrix multiplication unit works. Blue encodes the A molecules concentration, green encodes the output molecules concentrations, color intensities represent the concentration magnitude. In this realization B molecules can flow only in the outlets while A molecules freely diffuse from inlets to intermediates.

For clearness of presentation, the compartments were reorganized.

The first inlet is the topmost compartment and the second inlet the bottom most one. The row in the middle are outlets and intermediates are the connecting compartments between inlet and outlet. At the beginning of the computation (cf. Figure 2(a)), the input is encoded into the inlet concentrations (blue). If the reaction is slow compared to diffusion,^12^ the initial concentrations diffuse between inlet and intermediate, so that the concentrations inside one inlet and its respective intermediates is the same (cf. Figure 2(b)). The reaction in the intermediates continuously removes A molecules from the intermediates and converts them to B , which can then diffuse unidirectionally to the outlet. As the reaction is slow compared to the diffusion, even in the presence of a reaction in the intermediates belonging to the same inlet they still have roughly the same concentration as their respective inlet. If furthermore the reaction rate is the same inside all intermediates, the amount of B produced per unit time in a given compartment can only depend on its volume, i.e., for the same concentration and reaction rate a larger compartment produces more B molecules per unit time, than a smaller one. This is shown in Figure 2(c). Over time, more and more B molecules (green) accumulate in the outlets. Since the intermediates volumes of (1,2) and (2,2) are both larger than the ones of (1,1) and (2,1), respectively, a larger fraction of the molecules initially placed in both inlets end up in outlet 2 (hence the more intense green color in that outlet). The fact that the outlets are the smallest volumes in the example demonstrates how signal amplification is performed in the matrix multiplication unit: Forcing the same number of molecules into a smaller volume allows an increase in concentration and thus an amplification factor. It was shown in,^12^ that the concentration in the *j*-th outlet can be written as

1 $$C_{\mathrm{out},j}^{B,\mathrm{fin}}=\overset{2}{\underset{i=1}{\sum }}\chi_{ij}G_{i}C_{\mathrm{in},i}^{A,\mathrm{init}}$$

with $\chi_{\mathrm{ij}}=\frac{V_{\mathrm{ij}}}{\overset{2}{\underset{j=1}{\sum }}V_{\mathrm{ij}}}$ and $G_{i}=\frac{V_{\mathrm{in}}^{i}}{V}$ , where *V* indicates the outlet volume, *V*_*ij*_ are the intermediate compartments volumes and *V*_in_^*i*^ are the inlet volumes. Collecting the terms for all inlets and outlets in vectors, this equation can be written in the form

2 $$C_{\mathrm{out}}={\mathrm{MC}}_{\mathrm{in}}$$

where $C_{\mathrm{out}}$ represents the column vector collecting $C_{\mathrm{out},j}^{B,\mathrm{fin}}$ terms, $C_{\mathrm{in}}$ is the column vector collecting input $C_{\mathrm{in},i}^{A,\mathrm{init}}$ concentrations and M is the matrix we want to use, with *M*_*ji*_ = χ_*ij*_ *G*_*i*_. It was shown in ref 12 that this allows realization of an arbitrary positive valued matrix. In the previous paragraphs, the fundamental idea behind the computing structure was illustrated. The derivations provided in ref 12 are formulated in terms of ordinary differential equations (ODEs), which is justified by assuming homogeneous concentration distributions in each compartment. In what follows, we will first present a more accurate description in terms of partial differential equations (PDEs), which also account for inhomogeneous distributions of molecules. We will then use these distributions to obtain a thermodynamic perspective on the overall system.

From this point onward, we assume a slightly different realization of the matrix multiplication unit, involving two chemical reactions and different types of membranes to allow for the unidirectional flow of molecules.

The primary distinction between this work and previous studies on the computing unit is our adoption of the complete formalism of reversible reactions. We choose reactions that can be approximated as fully shifted toward the products while still maintaining nonzero backward reaction rates, ensuring consistency in the thermodynamic treatment.

In the original formulation presented by Angerbauer et al. and briefly discussed in the previous paragraphs, the unidirectional flow of molecules from intermediate compartments to outlets was entirely controlled by selectively permeable membranes placed in the connecting channels. Although membrane-controlled molecular flow systems exist in nature,^13^ their experimental implementation is challenging and requires precise tuning of micro- or nanostructured components.

In contrast, the present realization assumes that final reaction products cannot revert from outlets to intermediates, introducing the reaction B⇋C rather than forcing B molecules to flow exclusively into the outlets. From a mathematical perspective, this assumption preserves the design of the matrix computing unit and ensures the validity of eqs 1 and 2. However, in terms of practical implementation and ease of simulation, it significantly simplifies the problem. For instance, converting smaller B molecules into larger C molecules in the outlets and employing a membrane that prevents backflow based solely on molecular size is considerably simpler than designing a chemically selective permeable membrane. A depiction of the simulated architecture is provided in Figure 3. From a thermodynamic point of view, the computing unit can be described as a closed system in thermal equilibrium with a large heat reservoir. We assume that there are no significant temperature gradients at the boundaries between the matrix structure and the reservoir.

**Figure 3:**
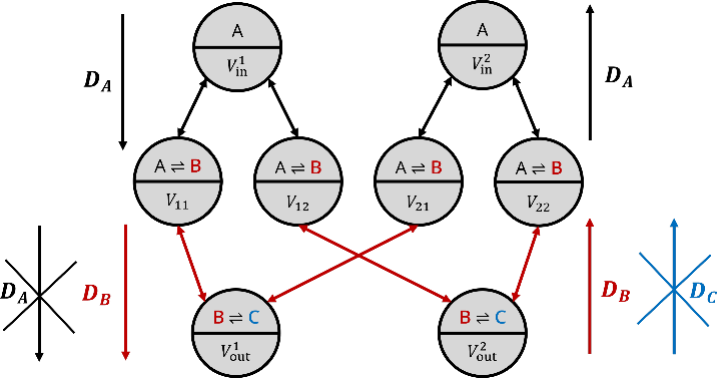
Schematic depiction of the system topology. The main difference from Angerbauer et al.^12^ is that a different set of reactions and membrane conditions is employed to achieve the same results. These differences are highlighted in the reaction schemes inside each compartment and in the arrows alongside the system topology. We assume that B and C molecules cannot flow back inside *V*_in_^*i*^ and *V*_*ij*_ respectively, while we assume that A molecules cannot flow toward *V*_out_^*j*^.

Within the framework of the Local Equilibrium Hypothesis,^9,14^ it has been shown that the time evolution of a thermodynamic system can be described by balance equations of the form

3 $$\frac{\partial s\left(r,t\right)}{\partial t}+\nabla \cdot J_{s}=\sigma \left(\left\{\varphi_{i}\right\},r,t\right)$$

where *s*(**r**,*t*) is the entropy density (assumed sufficiently smooth in space at each time *t*), **J**_s_ is the entropy flux across an infinitesimal surface element in the system, {φ_*i*_(**r**,*t*)} is the set of concentrations for all the involved chemical species and σ is the entropy production rate due to irreversible processes such as diffusion and chemical reactions.

In pioneering works by Onsager, de Groot, Prigogine, and others, it was demonstrated that under local equilibrium (and its generalizations), σ can be written as a sum of products of thermodynamic forces and flows^9,14–17^

4 $$\frac{\partial s\left(r,t\right)}{\partial t}+\nabla \cdot J_{s}=\underset{k}{\sum }F_{k}J_{k}$$

where *F*_*k*_ and *J*_*k*_ denote thermodynamic forces and flows, respectively.

A further important result is that, in accordance with the second law of thermodynamics, σ for an isolated system cannot be negative for spontaneous processes, even under nonequilibrium steady states. However, a general nonequilibrium extremum principle for the time evolution of σ has yet to be established. Any claim of such a principle in the past 50 years has been shown to admit at least one counterexample,^18,19^ limiting the direct application of eq 4 to cases where it can be explicitly solved for given boundary conditions or under the assumption that the system asymptotically relaxes to equilibrium. Still, under stronger constraints and in proximity to equilibrium, certain variational principles can be derived.^20–22^ When the system deviates only slightly from equilibrium, flows can be approximated as linear combinations of the forces, leading to

5 $$\sigma \left(r,t\right)=\underset{k}{\sum }F_{k}J_{k}=\underset{k}{\sum }\underset{i}{\sum }L_{ik}F_{k}F_{i}$$

where *L*_*ik*_ are phenomenological coefficients. In this linear regime, Onsager’s reciprocal relations apply,^15^ as will be exploited next.

Rewriting the matrix structure in terms of forces and flows requires fulfilling the conditions proposed by Angerbauer et al.^12^ for Fickian diffusion in the presence of chemical reactions

6 $$J_{i}=-D_{i}\nabla \varphi_{i}$$

and for any reaction,

7 $$J_{\alpha }={\left\{R_{f}-R_{b}\right\}}_{\alpha }$$

where the index *i* runs over the chemical species involved and α runs over the various chemical reactions, while *R*_*f*_ and *R*_*b*_ are the forward and backward reaction rates, respectively.

The requirement for Fickian diffusion imposes stringent constraints on the choice of chemical species, namely that any cross-coupling between different chemical potential gradients must be negligible. In contrast, the more general phenomenological laws of thermodynamics (cf. eq 5) would allow for coupling of different forces via the coefficients *L*_*ik*_.

In the Supporting Information, we provide a derivation, based on Prigogine’s treatment, detailing the conditions for Fickian diffusion and preventing back-diffusion of products. Here, we offer a concise summary. Let **I**_Ω_(**r**) be the indicator function over a domain Ω (e.g., a compartment of the matrix). We then assume that the diffusion coefficient *D*_*i*_ for the *i*-th species has the form

8 $$D_{i}\left(r\right)=I_{\Omega }\left(r\right)\cdot D_{i}$$

implying that in regions where diffusion is allowed, the diffusion coefficient remains constant, while it becomes zero in regions bounded by membranes. This approach captures selective diffusion of chemical species and blocks back-diffusion of products from intermediates and outlets to the inlets.

Similarly, for each elementary reaction of the form

9 A⇋B

we define

10 $$k_{j}^{\alpha }\left(r\right)=I_{\Omega }\left(r\right)\cdot k_{j}$$

11 $$R_{j}^{\alpha }=k_{j}^{\alpha }\left(r\right)\varphi_{i}$$

where *k*_*j*_ is the reaction rate constant in the domain Ω, α indexes each reaction, *j* denotes the forward (*f*) or backward (*b*) rate, and φ_*i*_ is the concentration of the relevant species.

For a single reaction, we might write

12 $$R_{f}^{1}=k_{f}^{\alpha }\left(r\right)\varphi_{A}$$

Note that here we treat each reaction as if it were unimolecular; however, the same reasoning holds if the reaction is bimolecular and one reactant is in large excess in the intermediate compartments.

Under these assumptions, we obtain the following pseudo-Fickian reaction–diffusion equations

13 $$\frac{\partial \varphi_{i}}{\partial t}=D_{i}\nabla^{2}\varphi_{i}+\underset{\alpha }{\sum }{\left\{R_{f}-R_{b}\right\}}_{\alpha }$$

and, as shown in the Supporting Information, if cross-diffusion coefficients can be neglected, the entropy density evolution can be written as

14 $$\frac{\partial s\left(r,t\right)}{\partial t}=\sigma_{\mathrm{diff}}+\sigma_{R}$$

where

15 $$\sigma_{\mathrm{diff}}=\frac{R}{T}\overset{3}{\underset{i=1}{\sum }}D_{i}\frac{{\left|\nabla \varphi_{i}\right|}^{2}}{\varphi_{i}}+\frac{2R^{2}}{T^{2}}\underset{i<k}{\sum }L_{ik}\frac{\nabla \varphi_{i}\cdot \nabla \varphi_{k}}{\varphi_{i}\varphi_{k}}\approx \frac{R}{T}\overset{3}{\underset{i=1}{\sum }}D_{i}\frac{{\left|\nabla \varphi_{i}\right|}^{2}}{\varphi_{i}}$$

and

16 $$\sigma_{R}=R\underset{\alpha }{\sum }{\left\{\left(R_{f}-R_{b}\right)\;\ln\left(\frac{R_{f}}{R_{b}}\right)\right\}}_{\alpha }$$

Here, *D*_*i*_ and *R*_*j*_ are as in eqs 8 and 11, while σ_diff_ represents the entropy production due to diffusion, and σ_R_ corresponds to the entropy production due to reactions. The term φ_*i*_ denotes the concentration of species *i*, whereas *v*_*i*_ is the partial molar volume of species *i*. The temperature is indicated by *T*, and ∇φ_*i*_ represents the gradient of the concentration of species *i*. Additionally, *R*_*f*_ and *R*_*b*_ refer to the forward and backward reaction rates, respectively.

Equations 14, 15, and 16 thus describe the total entropy production from both diffusive and reactive processes. Under the hypothesis that a stable equilibrium for the reaction-diffusion system exists, a number of results regarding the asymptotic behavior toward equilibrium are known. In particular, it is possible to show the exponential convergence to equilibrium according to the Entropy–Entropy-Dissipation inequality of Fellner et al.^23^ These analytical results on the matrix computing unit will be addressed after the thermodynamics analysis.

Although eq 3 is formally valid whenever each force and flow is taken into account, it is clear that solving it for a general system necessitates setting up a set of coupled mass-balance equations for the time evolution of each concentration variable.

Under our assumptions, this corresponds to defining the Reaction–Diffusion system in eq 13. We therefore employed the FiPy package^24^ and the gmsh meshing software^25^ to iteratively solve both sets of equations, approximating activity coefficients as constant whenever possible. In the Supporting Information, we provide a detailed description of the meshing procedure and a Google Colab script for the numerical integration of eqs 13 and 14 in our domains. The simulated matrix has been assumed to always start from a static configuration where the inlets *V*_in_^1^, *V*_in_^2^ (bottom left and top right squares in Figure 4) generate the inflow of molecules toward the intermediate compartments and, subsequent to the reactions, the outflow of B molecules toward the outlets *V*_out_^1^, *V*_out_^2^ (top left and bottom right squares in Figure 4).

**Figure 4:**
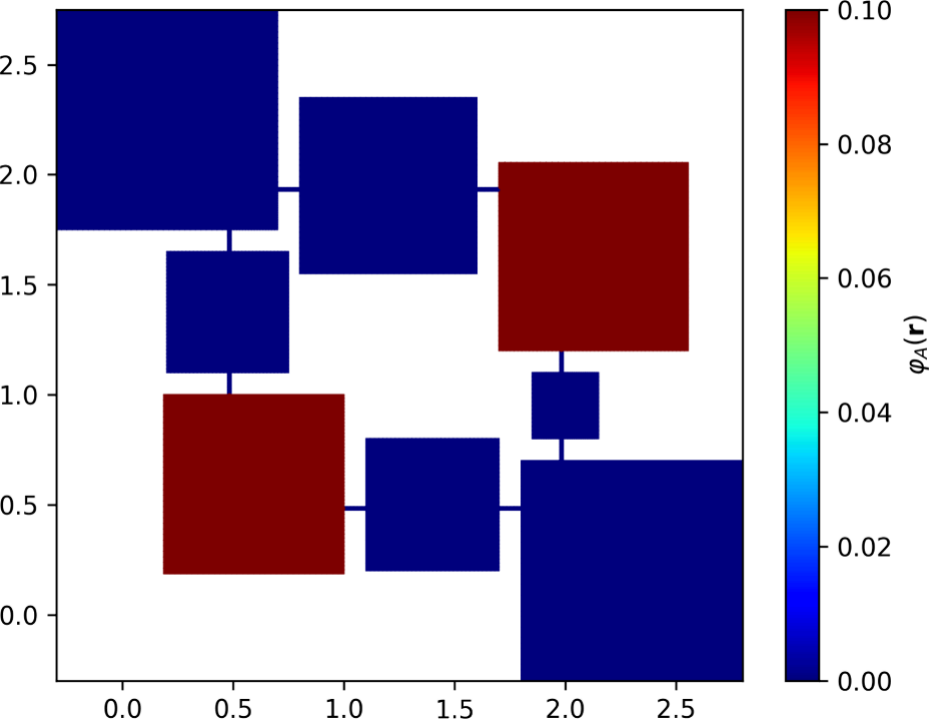
Distribution of A molecules at *t* = 0, $\varphi_{A}\left(r,0\right)$ , under assumptions [1]. The image is color coded to show the initial configuration for our system, red indicates higher concentration values, while blue represents zero concentration values.

In the following, we report the results of our modeling approach under three different limiting assumptions.

1 First, we solve eq 14 on a 2D mesh under the assumptions of infinite dilution, homogeneous distributions of A molecules in the inlets and sharp membranes.
2 Next, we consider a more realistic scenario in which we inject an inhomogeneous distribution (a Gaussian pulse) of A molecules into the first inlet, while preserving the same total number of molecules as in the homogeneous distribution case.
3 We then relax the conditions on *D*(**r**) by employing sigmoidal gate functions centered at the membrane boundaries.^26^

Once we solve for the space-dependent concentrations and entropy density, a straightforward integration over the relevant compartments yields both the outlet concentrations and the total entropy within the structure. The entropy as a function of time is given by

17 $$S\left(t\right)=\int_{V}s\left(r,t\right)\;dr$$

In each script, we use the following matrix multiplication

18 $$\left(\begin{array}{c} c_{\mathrm{out}}^{1}\\ c_{\mathrm{out}}^{2} \end{array}\right)=\left(\begin{array}{cc} M_{11} & M_{12}\\ M_{21} & M_{22} \end{array}\right)\left(\begin{array}{c} c_{\mathrm{in}}^{1}\\ c_{\mathrm{in}}^{2} \end{array}\right)=\left(\begin{array}{c} 0.09725\\ 0.045 \end{array}\right)=\frac{1}{100}\left(\begin{array}{cc} 30.25 & 64\\ 36 & 9 \end{array}\right)\left(\begin{array}{c} 0.1\\ 0.1 \end{array}\right)$$

In principle, we can obtain the total entropy at *t* = 0 by assuming a constant concentration of 0.1 in the inlets. However, because our main objective is to study the qualitative behavior of entropy, we set the reference state at *t* = 0 to have zero entropy density. Nonetheless, our scripts still produce a value for *S*(0), which depends on the time step chosen for integrating the equations. Readers should be aware that, even under our assumptions, the reliability of *S*(0) is strongly affected by the selected time step.

All simulations were performed under the assumption that, because the solvent is in large excess, partial molar volume changes are negligible. If needed, one may still determine the relevant ratios and modify the equations to ensure positivity (without loss of generality), as long as we consider mixtures of nonionic organic molecules.

The simulation parameters and an example set of chemicals for realizing the system are summarized in Table 1.

**Table 1:** Summary of Possible Reactants, Diffusion Coefficients, and Reaction Constants

species	*D* (μ m^2^/s)	role	*k*_f_ (s^–1^)
Formaldehyde (CH_2_O)	1900	Reactant A	1.0
Hydroxymethylphenol (C_7_H_8_O_2_)	1300	Reactant B	0.1
Tannic acid adduct	80	Product C	0.1

Under assumptions [1] (homogeneous φ­(**r**,0) and sharp *D*(**r**)), the numerical integration results show that, given a suitable meshing of a 2D matrix multiplication unit and starting from homogeneous inlet concentrations (Figure 4), the asymptotic behavior of the outlet species concentration reaches the predicted values in about 200 s (Figures 5 and 6). The entropy increases monotonically until it converges to a steady-state value (Figure 7).

**Figure 5:**
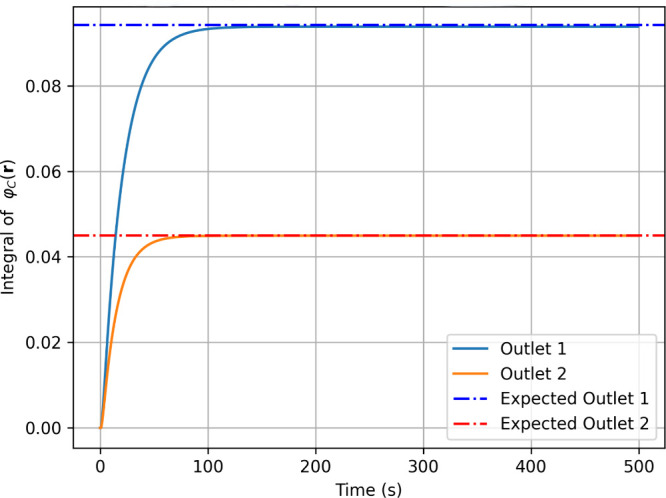
Time evolution of the C molecule concentration integrated over outlets 1 (orange), and 2 (blue) using linearly scaled timesteps in FiPy. Dashdot lines represent expected output values.

**Figure 6:**
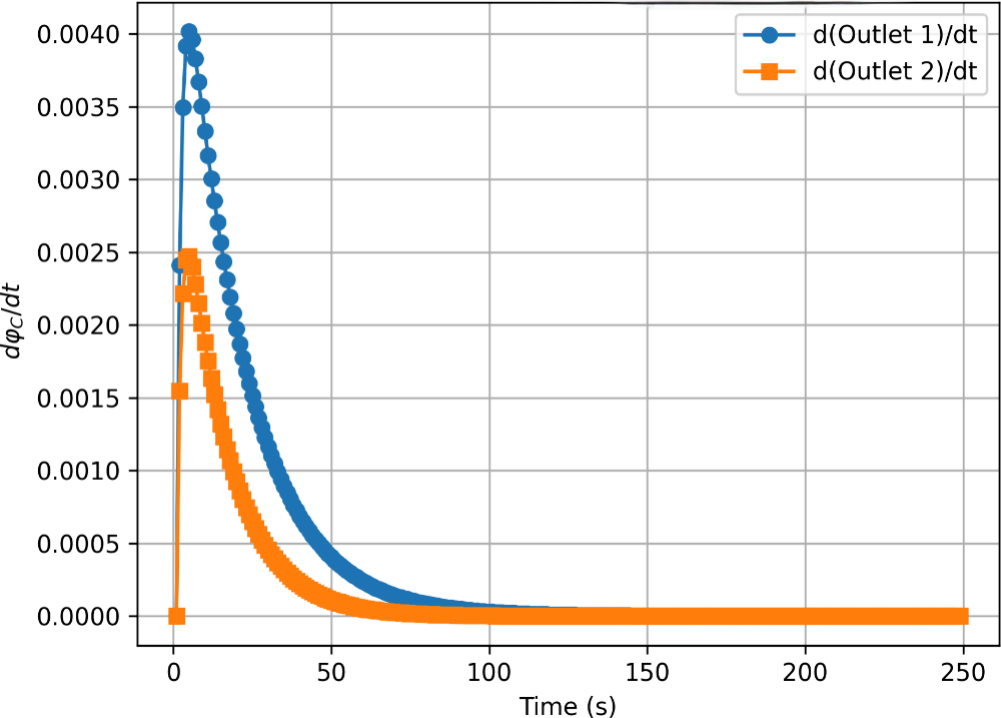
First derivative of the total outlet concentration ( $\int \varphi_{C}dr$ ) over time, indicating convergence after approximately 150 s.

**Figure 7:**
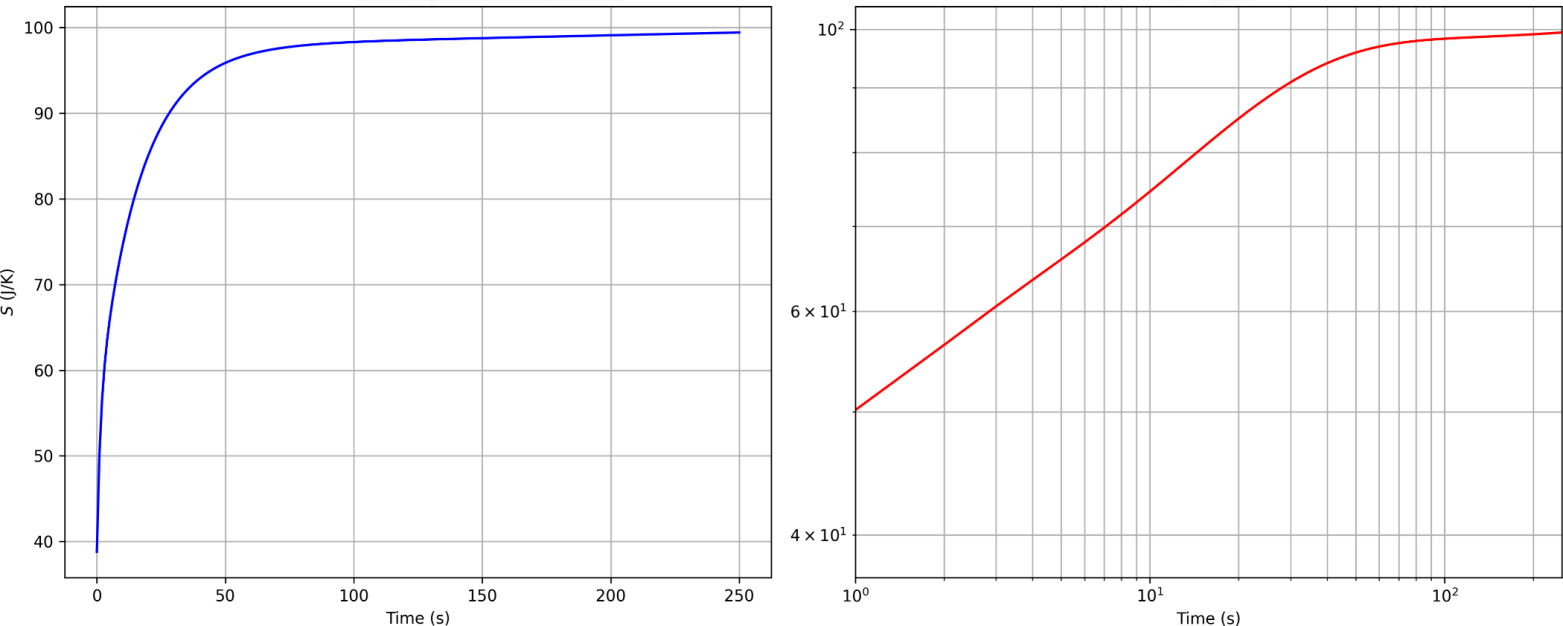
Comparison of the linear (left) and log–log (right) plots of *S*(*t*) under the assumption of homogeneous initial distributions of molecules in both inlets.

Figure 6 shows the first derivative of the total outlet concentration (φ_C_) over time, indicating that the computation converges to a nearly constant value after about 200 s, confirming that the system is close to steady state.

Regarding entropy, Figure 7 shows a monotonically increasing trend, in agreement with the physical requirement of positive entropy production. In addition, during the initial stages of the simulation a linear log–log relation is found, suggesting a power-law type of scaling later modulated to converge to the steady state. The temporal evolution of the entropy density is depicted in more detail in a short video available in the Supporting Information.

Under the assumption of perfectly sharp membranes and negligible cross-coupling of diffusive flows—i.e., in the ”naive” dimensioning limit^8^ —the matrix computation spontaneously converges. However, we cannot guarantee *a priori* that the same behavior would occur if the simulation were initialized with a spatially inhomogeneous distribution of A molecules (i.e., under assumptions [2]).

Nevertheless, assuming reasonably large $D_{A}$ values, even starting from such a distribution at the first inlet (a bivariate Gaussian pulse,^27^ depicted in Figure 8) leads to qualitatively and quantitatively similar output values (Figure 9). Furthermore, Figure 10 shows that the total entropy produced under assumptions [2] is comparable to that under assumptions [1], although it is slightly lower in value.

**Figure 8:**
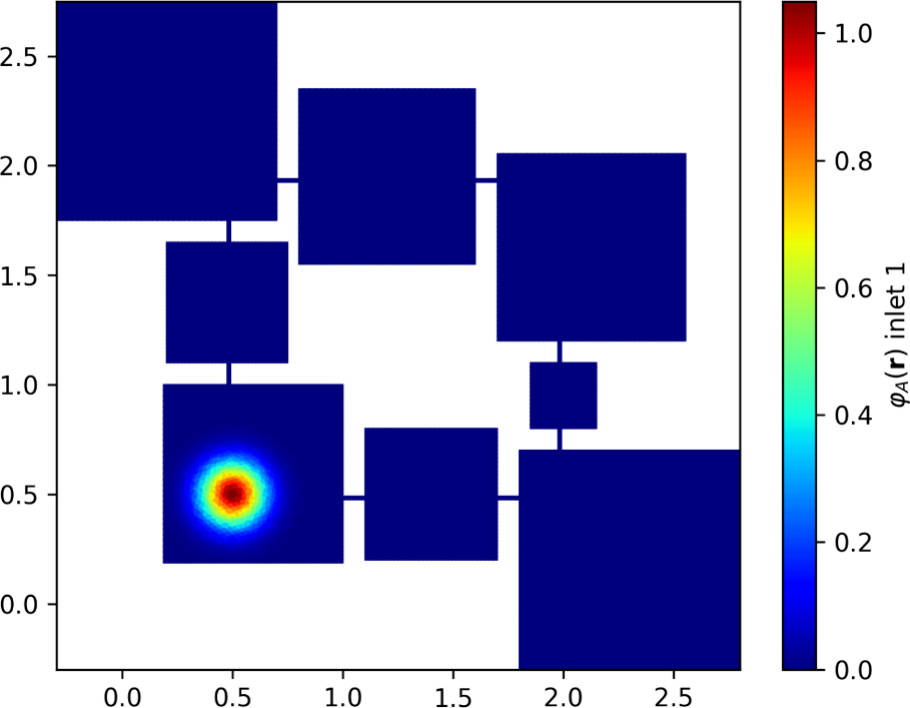
Inhomogeneous distribution of A molecules, φ_A_(**r**, 0), at inlet 1 for *t* = 0. The depiction is color-coded, with red indicating higher concentration values and blue indicating lower ones.

**Figure 9:**
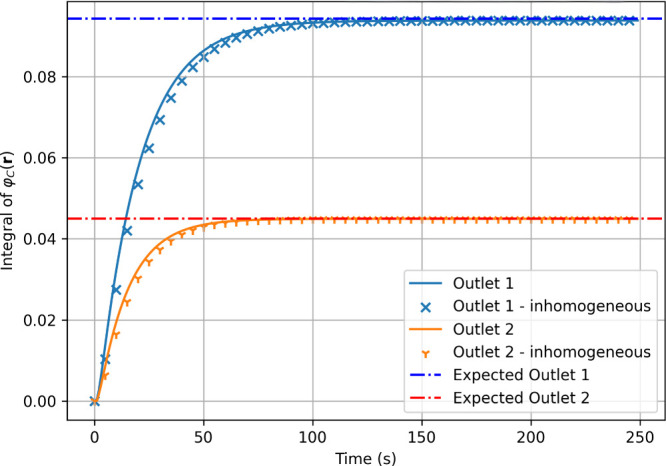
Time evolution of the C molecule concentrations in different outlets under homogeneous (solid lines) and inhomogeneous (scatter plots) initial conditions.

**Figure 10:**
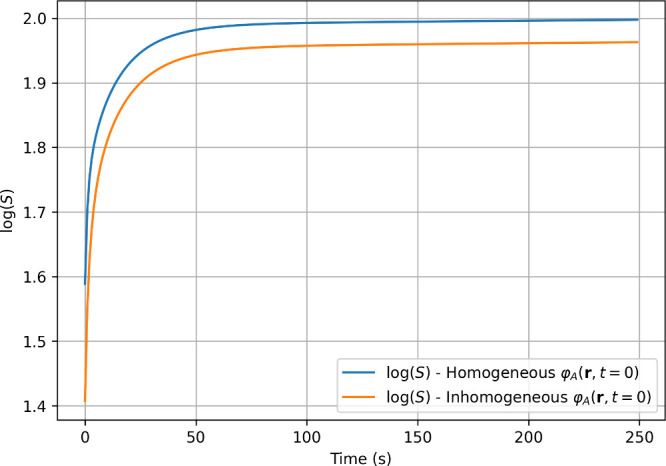
Time evolution comparison of *S*(*t*) on a semilog scale for the inhomogeneous (orange line) and homogeneous (blue line) initial conditions.

To better compare the time evolution of concentrations under assumptions [1] and [2], we also plotted the derivatives of $\int \varphi_{C}\;dr$ over time (Figure 11), finding that in the limit of large times, they relax to zero under both initial conditions with minimal differences.

**Figure 11:**
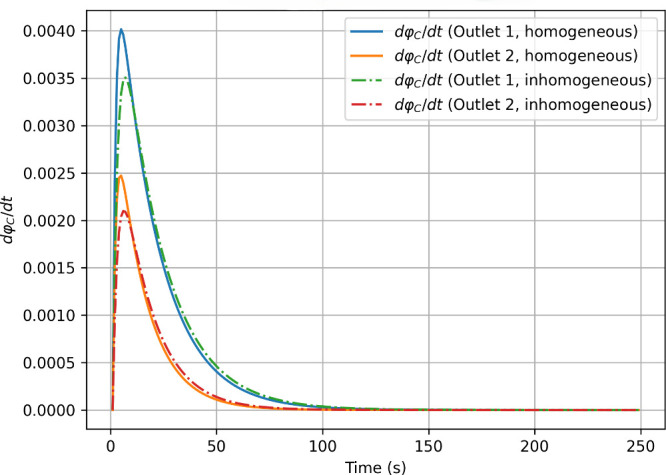
Comparison of the concentration integrals first derivatives in Outlets 1 and 2 starting from homogeneous (solid lines) and inhomogeneous (dashdot lines) $\varphi_{A}\left(r,t\right)\in \mathrm{inlet}1$ .

Having shown that relaxing the requirement for homogeneous inlet concentrations is not problematic (provided we use perfectly soluble molecules), we now ask whether relaxing the assumption of sharp diffusion coefficients (i.e., using assumptions [3]) produces a similar effect. For this purpose, we employed spatially varying diffusion coefficients modeled by sigmoidal gates centered at the membrane boundaries, each with a stiffness parameter *f*. The construction of these functions is detailed in Section S2 of the Supporting Information.

Our simulations indicate that while smooth *D*(**r**) functions are more general than sharp membranes, a key requirement for achieving the correct convergence is that the “steepness” of the sigmoidal gates remains sufficiently large to avoid excessive loss of C molecules from the outlets to intermediate compartments. This is evident by comparing the results from sharp *D* values with those obtained using smooth *D* functions (Figure 12 for concentrations and Figure 13 for entropy). In particular, we observe that in the limit of small times and for smooth diffusion coefficients the log–log entropy plot insets do not show signs of a simple linear scaling. This observation, that is unrelated to the convergence of concentrations to their equilibrium values, suggests that the power law scaling is not recovered lifting the assumption of sharp membranes. Moreover, employing sigmoidal gates slightly increases the steady-state entropy value for sufficiently large *f* values, which is intuitively reasonable because allowing C molecules to diffuse across a larger portion of the mesh expands the number of microstates accessible to the system. In terms of system design, one has tho choose the smallest *f* value that guarantees a suitable convergence criterion to the expected output. This is, in general a form of constrained optimization, since sharper membranes are generally more difficult to manufacture but lead to more accurate results.

**Figure 12:**
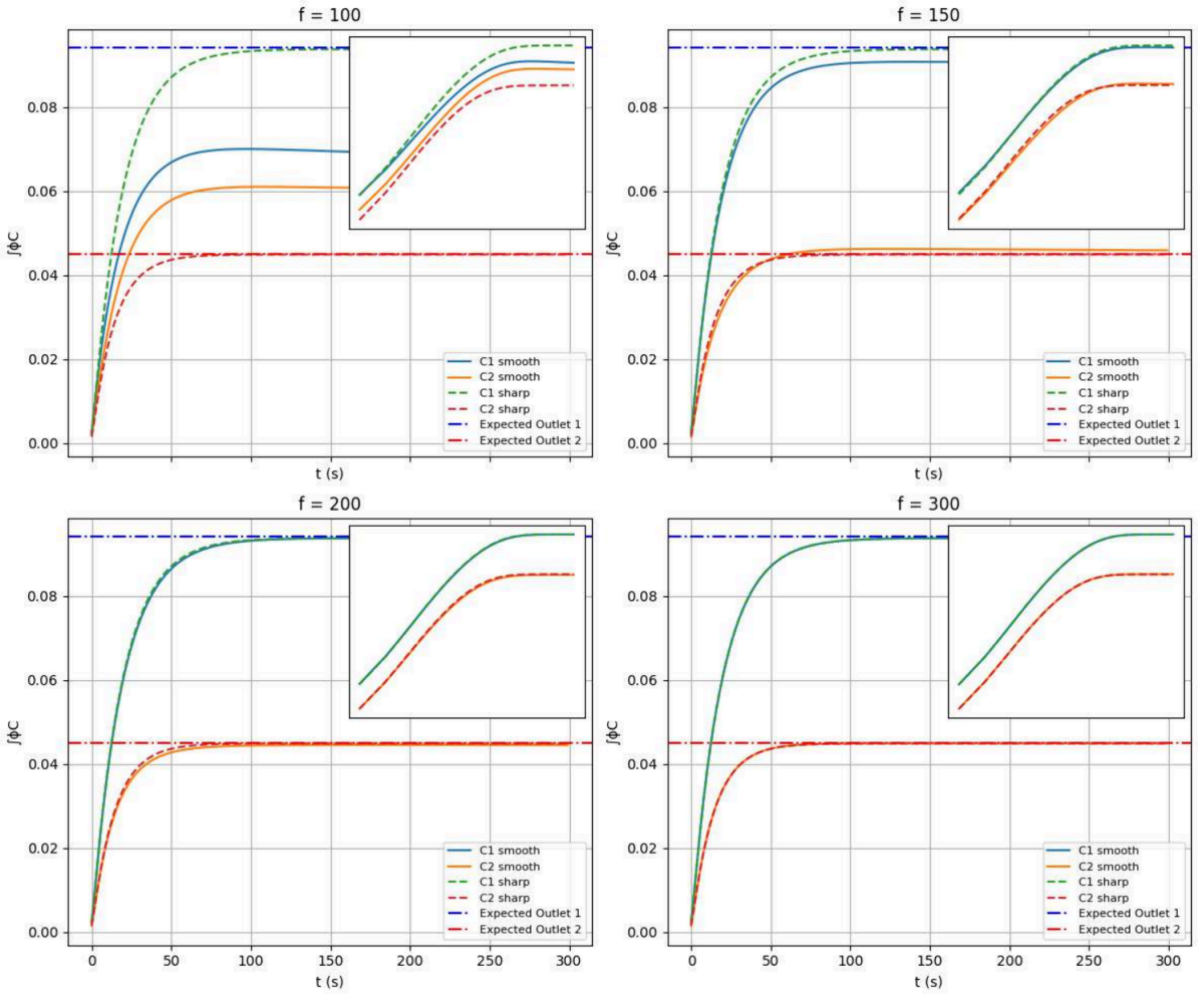
Comparison of simulations with and without smooth *D*(**r**) functions for different *f* values. Continuous lines represent sharp-membrane results, while scatter points indicate sigmoidal-gate results. Inlets show the same results in log–log scale.

**Figure 13:**
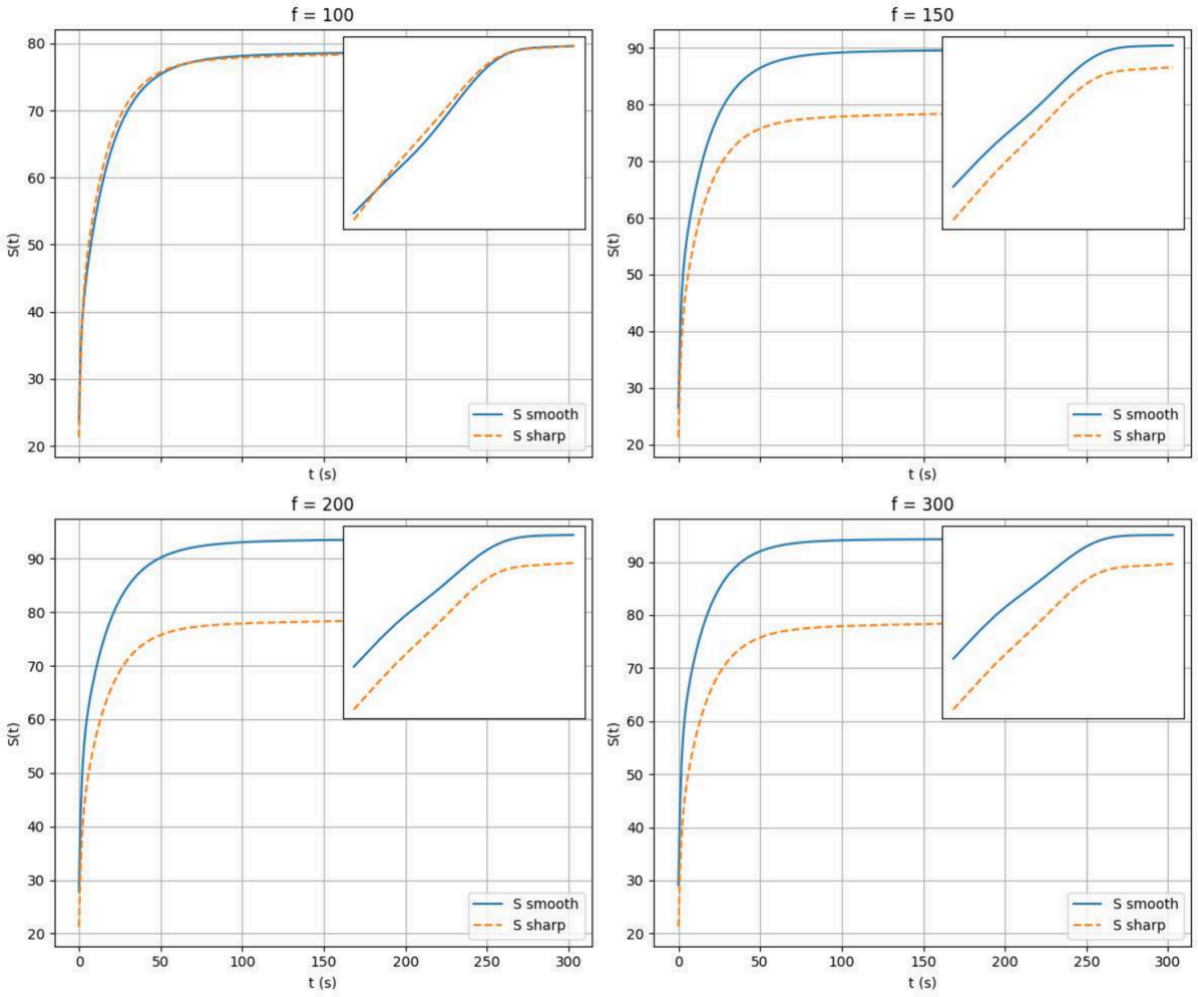
Comparison of the entropy evolution with and without smooth *D*(**r**) functions for different *f* values. Orange lines indicate sharp-membrane results, while blue lines show sigmoidal-gate results. Insets show the same results in log–log scale.

To support the numerical results, we will now establish rigorously that the reaction-diffusion realization of the matrixcomputing unit introduced in^12^ converges exponentially fast to its unique equilibrium, by invoking the entropy method of Fellner.^23^ Our presentation is self-contained and highlights precisely the hypotheses required on the diffusion coefficients, reaction network, and spatial domain.

Let $\Omega \subset R^{n}$ be a bounded, connected Lipschitz domain (the set of compartment and channels). In general, it is provable that for any *I* × *J* matrix, the associated Ω domain and its (sufficiently smooth) border satisfy the hypotheses of Fellner’s theorem. More rigorous arguments are given in the Supporting Information. We denote by φ_*i*_(**r**,*t*), *i* = 1,2,3, the concentrations of species A,B,C respectively, evolving according to a rewritten version of eq 13

19 $$\frac{\partial \varphi_{i}}{\partial t}=\nabla \cdot \left(D_{i}\left(r\right)\nabla \varphi_{i}\right)+\overset{3}{\underset{j=1}{\sum }}a_{ij}\varphi_{j},\;\partial_{\nu }\varphi_{i}{|}_{\partial \Omega }=0$$

with nonnegative initial data of total mass

$$M=\overset{3}{\underset{i=1}{\sum }}\int_{\Omega }\varphi_{i}\left(r,0\right)\;dr>0$$

Here

- *D*_*i*_(**r**) ≥ 0 are piecewiseconstant diffusion coefficients, with at least one index *i* such that *D*_*i*_ ≡ *d*_min_ > 0 on a subset of Ω that *percolates* in the sense of ref 23.
- The reaction matrix *A* = (*a*_*ij*_) satisfies *a*_*ij*_ ≥ 0 for *i*≠ *j*, *a*_*ii*_ = −∑_*j*≠ *i*_ *a*_*ji*_, and is irreducible (so that the directed graph *i* → *j* whenever *a*_*ji*_ > 0 is strongly connected).

By Perron-Frobenius there exists a unique positive equilibrium

20 $$X_{\infty }=\left(\varphi_{1,\infty },\varphi_{2,\infty },\varphi_{3,\infty }\right)>0,\;\overset{3}{\underset{i=1}{\sum }}\varphi_{i,\infty }=M$$

solving *A X*_∞_ = 0.

We introduce the Fellner quadratic relativeentropy functional

21 $$E\left(t\right)=\overset{3}{\underset{i=1}{\sum }}\int_{\Omega }\frac{{\left(\varphi_{i}\left(r,t\right)-\varphi_{i,\infty }\right)}^{2}}{\varphi_{i,\infty }}\;dr$$

A direct computation (cf. Lemma 2.3 in ref 23) leads to the entropy dissipation equation

22 $$-\frac{d}{dt}E\left(t\right)=D_{\mathrm{diff}}\left(t\right)+D_{\mathrm{react}}\left(t\right)$$

where the *diffusive* part is

$$D_{\mathrm{diff}}\left(t\right)=2\overset{3}{\underset{i=1}{\sum }}\int_{\Omega }D_{i}\left(r\right)\frac{|\nabla \left(\varphi_{i}-\varphi_{i,\infty }\right){|}^{2}}{\varphi_{i,\infty }}\;dr$$

and the *reactive* part is

$$D_{\mathrm{react}}\left(t\right)=\underset{1\leq i<j\leq 3}{\sum }\left(a_{ji}\varphi_{i,\infty }+a_{ij}\varphi_{j,\infty }\right)\int_{\Omega }{\left(\frac{\varphi_{i}}{\varphi_{i,\infty }}-\frac{\varphi_{j}}{\varphi_{j,\infty }}\right)}^{2}\;dr$$

Both those results are, in a sense, similar to the entropy production contributions we derived based on Prigogine’s Linear Non-Equilibrium Thermodynamics. In the Supporting Information, we explicitly derive an analogous entropy density functional which behaves like Fellner’s, thus showing the connection between the classic theory of local-equilibrium thermodynamics and the elegant treatment of the more general CRN theory. We now invoke the two main coercivity estimates proved in.^23^

*(i) Diffusive Coercivity.* Since Ω is connected Lipschitz, its Neumann Laplacian has spectral gap λ_1_ > 0. For each *i* with *D*_*i*_ ≥ *d*_min_ > 0, Poincaré’s inequality yields

$$\int_{\Omega }d_{i}\left(r\right)|\nabla v{|}^{2}\;dr\geq d_{\min}\lambda_{1}\int_{\Omega }v^{2}\;dr,v=\frac{\varphi_{i}-\varphi_{i,\infty }}{\sqrt{\varphi_{i,\infty }}}$$

and summing over the diffusive indices gives

23 $$D_{\mathrm{diff}}\left(t\right)\geq 2d_{\min}\lambda_{1}\underset{i:d_{i}>0}{\sum }\int_{\Omega }\frac{{\left(\varphi_{i}-\varphi_{i,\infty }\right)}^{2}}{\varphi_{i,\infty }}\;dr\geq \Lambda_{\mathrm{diff}}E\left(t\right),\;\Lambda_{\mathrm{diff}}=\frac{2}{3}d_{\min}\lambda_{1}$$

*(ii) Reactive Coercivity*. Irreducibility of *A* implies (by Lemma 2.4 in ref 23) that there exists μ > 0 such that

24 $$D_{\mathrm{react}}\left(t\right)\geq \mu E\left(t\right)$$

Combining [Disp-formula eq22], 23, and 24 yields the entropy–entropy dissipation estimate

$$-\frac{d}{dt}E\left(t\right)=D_{\mathrm{diff}}\left(t\right)+D_{\mathrm{react}}\left(t\right)\geq \left(\Lambda_{\mathrm{diff}}+\mu \right)E\left(t\right)=\Lambda E\left(t\right),\Lambda >0$$

By Grönwall’s lemma,

E(t)≤E(0)exp(−Λt)

which in turn implies

$${∥\varphi_{i}\left(\cdot ,t\right)-\varphi_{i,\infty }∥}_{L^{2}\left(\Omega \right)}^{2}\leq \varphi_{i,\infty }E\left(0\right)e^{-\Lambda t},i=1,2,3$$

Hence the computing unit converges exponentially fast to its unique equilibrium ([Disp-formula eq20]) under the precise assumptions stated above. There is, however, a strong difference between our system and the ones described by Fellner et al., that is, we admit, in the case of non-negative diffusion coefficients, that some of them might be zero in some subportions of Ω. In this case, as reported in their main text, it is still possible to invoke the same results, but it is not possible to give a simple estimate of the Λ constant. Under the smooth diffusion coefficient assumptions, due to the asymptotic convergence toward zero of the chosen sigmoidal functions, or just assuming that instead of zero, the relevant diffusion coefficients under ”sharp” membrane conditions are small but finite, we recover the conditions for Fellner’s theorem to hold, as evident from Figure 14, where in the limit of sharp coefficients, the functional ratio of D/E proves to be always larger than Λ_theory_, which was computed employing the results of ref 23 to be 2.3·10^–7^ s^–1^ c.ca.

**Figure 14:**
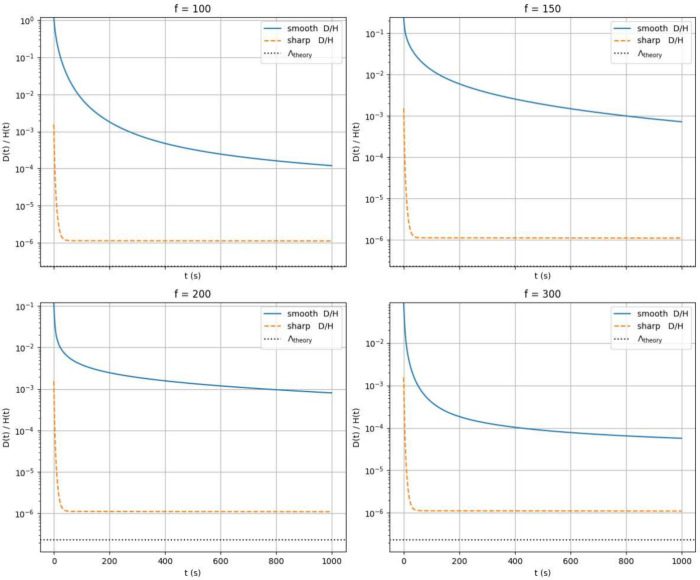
Comparison, in semilog scale, of the D/E ratio for different *f* values of the sigmoidal diffusion coefficients (blue line). For reference, we also report the same ratio under assumptions [1] (orange dash-line) and a black dotted line marking the theoretical Λ for our mesh.

Lastly, while our main text focuses on the 2 × 2 demonstration, practical deployment demands an understanding of how resources grow when we generalize to an *I* × *J* multiplier. Here we walk through each scaling dimensionhardware, speed, thermodynamic constraints, and configuration effortin a simplified manner building on the original results in ref 12. The reader should address the following discussion as a mere line of reasoning for further, more rigorous, studies to be made.

First, building an *I* × *J* array of reaction-diffusion cells requires one *inlet* for each of the *I* inputs, one *outlet* for each of the *J* outputs, and one *intermediate compartment* for every matrix entry. Altogether, the cell count is

25 $$N_{\mathrm{comp}}=I+IJ+J=O\left(IJ\right)$$

not including the two diffusion channels per intermediate (one for A -mixing, one for *B*-export). Thus, hardware cost grows quadratically in matrix dimensions.

Despite this, *compute latency* can remain constant because each inlet-intermediate-outlet chain operates fully in parallel. Within each chain, three processes compete:

26 $$\tau_{\mathrm{diff}}\approx \frac{a^{2}}{2D},\tau_{A\to B}\approx \frac{1}{k_{f_{B}}},\tau_{B\to C}\approx \frac{1}{k_{f_{C}}}$$

Here, *a* is the characteristic compartment size, *D* the diffusion coefficient, and *k*_*f*_*B*__,*k*_*f*_*C*__ the forward reaction rates. By fixing *a*, *k*_*f*_B__, and *k*_*f*_C__ independently of *I*,*J*, the overall time is

27 $$\tau_{\mathrm{total}}=\max\left\{\tau_{\mathrm{diff}},\tau_{A\to B},\tau_{B\to C}\right\}=O\left(1\right)$$

meaning the entire *I* × *J* multiply completes in constant wallclock time. However, thermodynamic consistency imposes a crucial bound: reactions must be slow enough that diffusion maintains nearly uniform concentrations. Concretely,

28 $$k_{f_{B}},k_{f_{C}}\ll \frac{D}{a^{2}}$$

If we instead pack all *IJ* cells into a fixed total volume *V*_tot_, each cell’s size shrinks as *a*∼(*V*_tot_/(*IJ*))^1/3^. Diffusion then accelerates τ_diff_ ∝ *a* ^2^/*D* ∝ (IJ)^−2/3^, and reaction rates can be raised to *k* ∼ *D*/*a* ^2^, but beyond that limit the time again scales like τ ∼ *a* ^2^/*D*. Finally, configuring an arbitrary weight matrix $M\in R^{J\times I}$ requires assigning each cell a volume proportional to its corresponding weight:

29 $$V_{i,j}=\left|M_{j,i}\right|,V_{\mathrm{in},i}=\overset{J}{\underset{j=1}{\sum }}\left|M_{j,i}\right|,V_{\mathrm{out},j}=1$$

which takes *O*(*IJ*) simple arithmetic operations. Selecting the two global rates *k*_f_,*k*_C_ is *O*(1) .

In summary, an *I* × *J* chemical multiplier demands *O*(*IJ*) fluidic hardware, delivers constanttime throughput under fixed cell dimensions, and obeys clear thermodynamic bounds on reaction speeds. Packing more cells into the same volume trades even faster operation (∝ (*IJ*)^−2/3^) against reduced precision and stricter fabrication constraints on gating.

*Conclusions*. In conclusion, we have assessed the thermodynamic conditions for a matrix-multiplication computing unit to operate under the local equilibrium hypothesis. Along with the standard conditions stated in the Supporting Information (Section S1), two key requirements are necessary to ensure both convergence to the desired output values and optimize entropy production: (a) cross-diffusion flows between different chemical species must be negligible, and (b) membranes must be sufficiently smooth to create gentle gradients at the compartment boundaries but sharp enough to avoid concentration leakages. To further refine our understanding of the asymptotic behavior for a general computing unit, we also investigated and proved the validity of Fellner’s theorem for both the ”sharp” and ”soft” membrane conditions. This analysis was carried out under the only requirement that the system topology remains unchanged by its geometrical realization.

We also showed that, in principle, any *I* × *J* matrix computation is *O*(1) with respect to latency, and scales quadratically in number of compartments.

Future work will include modeling electrically charged species and explicitly accounting for stochastic fluctuations in chemical composition arising from parasitic reactions and thermal noise at the nanoscale. Furthermore, investigations into the maximization of entropy production by varying system parameters using AI optimization strategies, as well as further refinements in modeling the injection dynamics of A molecules, are ongoing.

## Supplementary Material









## Data Availability

A .ipynb Google Colab script is available in the following GitHub repository.

## Notes

The mathematical derivation of the equations in the main text, along with a summary of the physical requirements for these equations to hold. This is followed by a description of the sigmoidal gates implementation and the meshing procedure (PDF)
A video illustrating the time evolution of entropy density (MP4)
A .ipynb Google Colab script (ZIP)
Transparent Peer Review report available (PDF)
