# Supplementary material for: Thermodynamics Analysis of a Reaction-Diffusion Matrix Multiplication Computing Unit under the Linear Non-Equilibrium Regime
Source: J Phys Chem Lett. 2025 Jun 13;16(25):6293–304. doi: 10.1021/acs.jpclett.5c00834 (PMC12207667; doi:10.1021/acs.jpclett.5c00834)
Supplement: Supplementary file 2 [file jz5c00834_si_002.pdf]

SUPPORTING INFORMATION FOR: Thermodynamics analysis of a Diffusion-Reaction Matrix Multiplication computing unit under the Linear Non-Equilibrium Regime

Giuseppe S. Basile,[†,‡] Stefan Angerbauer,[¶] Giuseppe Grasso,[†] Werner Haselmayr,[*,¶] and Nunzio Tuccitto[*,†]

†*Department of Chemical Sciences, University of Catania, Catania, Viale Andrea Doria 6, 95125, Italia*

‡*Scuola Superiore di Catania, Catania, Via Valdisavoia 9, 95123, Italia*

¶*Institute for Communication Engineering and RF-Systems, Johannes Kepler University Linz, Austria*

E-mail: werner.haselmayr@jku.at; nunzio.tuccitto@unict.it

Theoretical Framework

We derive the entropy production for a four-component system with solute concentrations $\varphi_1, \varphi_2, \varphi_3$ and solvent φ_4 (in large excess, $\varphi_4 \gg \varphi_1, \varphi_2, \varphi_3$), undergoing reversible reactions $1 \rightleftharpoons 2$ and $2 \rightleftharpoons 3$. The derivation assumes **ideal solutions** ($\gamma_i = 1$), **mechanical equilibrium** ($\nabla P = 0$), **isothermal conditions**, and **local incompressibility**:

$$\sum_{i=1}^{4} v_i \mathbf{J}_i = 0 \implies \mathbf{J}_4 = -\frac{1}{v_4} \sum_{i=1}^{3} v_i \mathbf{J}_i, \tag{1}$$

where v_i are partial molar volumes and $\mathbf{J}_i$ are molar fluxes in a volume-fixed frame. The solvent approximation ($\nabla\varphi_4 \approx 0$, $v_4 \gg v_1, v_2, v_3$) simplifies cross-coupling terms.

The total entropy production rate σ includes contributions from diffusion and reactions:

$$\sigma = \underbrace{\sum_{i=1}^{4} \mathbf{J}_i \cdot \mathbf{F}_i}_{\text{Diffusion}} + \underbrace{\frac{1}{T} \sum_{\alpha=1}^{2} A_\alpha \left\{R_f - R_b\right\}_\alpha}_{\text{Reactions}}, \tag{2}$$

where $\mathbf{F}_i = -\frac{1}{T}\nabla\mu_i$ is the diffusive thermodynamic force, and $A_\alpha = \sum_{i=1}^{4} \nu_{i\alpha}\mu_i$ is the affinity for reaction α. For ideal solutions, $\mu_i = \mu_i^0 + RT\ln\varphi_i$, leading to:

$$\mathbf{F}_i = -\frac{R}{T}\frac{\nabla\varphi_i}{\varphi_i} \quad (i = 1, 2, 3), \quad \mathbf{F}_4 = -\frac{R}{T}\frac{\nabla\varphi_4}{\varphi_4}. \tag{3}$$

Substituting $\mathbf{J}_i = \sum_{k=1}^{3} L_{ik}\mathbf{F}'_k$ (with $\mathbf{F}'_k = \mathbf{F}_k - \frac{v_k}{v_4}\mathbf{F}_4$) into the diffusion term, and using $\nabla\varphi_4 \approx 0$, the entropy production becomes:

$$\sigma_{\text{diff}} = \frac{R^2}{T^2} \sum_{i,k=1}^{3} L_{ik} \frac{\nabla\varphi_i}{\varphi_i} \cdot \frac{\nabla\varphi_k}{\varphi_k}. \tag{4}$$

Diagonal Onsager coefficients L_{ii} relate to Fickian diffusion coefficients via $\mathbf{J}_i = -D_i\nabla\varphi_i$:

$$L_{ii} = \frac{D_i\varphi_i}{R}. \tag{5}$$

Cross-terms L_{ik} ($i \neq k$) describe solute-solute interactions. Under the solvent approximation:

$$\sigma_{\text{diff}} \approx \frac{R}{T}\sum_{i=1}^{3} D_i \frac{|\nabla\varphi_i|^2}{\varphi_i} + \frac{2R^2}{T^2}\sum_{i<k} L_{ik}\frac{\nabla\varphi_i \cdot \nabla\varphi_k}{\varphi_i\varphi_k}. \tag{6}$$

For reactions $\alpha = 1, 2$, the affinity $A_\alpha = RT\ln\frac{R_{f\alpha}}{R_{b\alpha}}$ drives the net rate $w_\alpha = R_{f\alpha} - R_{b\alpha}$. The reaction entropy production is:[1]

$$\sigma_{\text{rxn}} = R\sum_{\alpha=1}^{2}\left\{w\ln\frac{R_f}{R_b}\right\}_\alpha. \tag{7}$$

Near equilibrium ($w_\alpha \ll R_{f\alpha}, R_{b\alpha}$), this simplifies to $\sigma_{\text{rxn}} \approx \frac{R}{R_{\text{eq}}}\sum_{\alpha=1}^{2} w_\alpha^2$. The total entropy production combines all terms:

$$\boxed{\sigma = \frac{R}{T}\sum_{i=1}^{3} D_i \frac{|\nabla\varphi_i|^2}{\varphi_i} + \frac{2R^2}{T^2}\sum_{i<k} L_{ik}\frac{\nabla\varphi_i \cdot \nabla\varphi_k}{\varphi_i\varphi_k} + R\sum_{\alpha=1}^{2}\left\{(R_f - R_b)\ln\frac{R_f}{R_b}\right\}_\alpha.} \tag{8}$$

Which for $i \neq k,\ L_{ik} = 0$, reduces to:

$$\boxed{\sigma = \frac{R}{T}\sum_{i=1}^{3} D_i \frac{|\nabla\varphi_i|^2}{\varphi_i} + R\sum_{\alpha=1}^{2}\left\{(R_f - R_b)\ln\frac{R_f}{R_b}\right\}_\alpha.} \tag{9}$$

A smooth function approach to describe membranes

A relatively simple way to model membranes is that of applying spatial restrictions on the behaviour of diffusion coefficients, in particular we would like to model them as $D = D(\mathbf{r})$, with $D \in \mathrm{C}^i$ where i is at least 1.

In the following we describe the methodology for constructing spatially varying diffusion coefficients in membrane modeling using sigmoid gate functions. These functions provide smooth spatial transitions and enable the dynamic adaptation of diffusion regions.

Given a membrane region R discretized into N grid cells with coordinates (x_i, y_i) for $i =$

$1, \ldots, N$, the centroid of the region is computed as

$$\bar{x} = \frac{1}{N}\sum_{i=1}^{N} x_i, \quad \bar{y} = \frac{1}{N}\sum_{i=1}^{N} y_i. \tag{10}$$

These centroid coordinates are used to define dynamic boundaries that adapt to the membrane geometry.

To spatially localize the diffusion coefficients, we use sigmoid gate functions. A general sigmoid gate is defined as

$$\Gamma_{\text{gate}}(u; u_0, f) = \frac{1}{1 + \exp\left[-f\,(u - u_0)\right]}, \tag{11}$$

where u is the spatial coordinate, u_0 is the boundary position (which may be fixed or derived from the region centroid), and f is the steepness parameter controlling the sharpness of the transition. The function in (11) is continuous and infinitely differentiable, ensuring smooth transitions. It is monotonically increasing with

$$\lim_{u\to-\infty} \Gamma_{\text{gate}}(u; u_0, f) = 0 \quad \text{and} \quad \lim_{u\to+\infty} \Gamma_{\text{gate}}(u; u_0, f) = 1. \tag{12}$$

Its derivative is given by

$$\frac{d}{du}\Gamma_{\text{gate}}(u; u_0, f) = f\,\frac{\exp\left[-f\,(u - u_0)\right]}{\left(1 + \exp\left[-f\,(u - u_0)\right]\right)^2} \tag{13}$$

which attains its maximum at $u = u_0$ and is symmetric about that point.

The diffusion coefficients D_1, D_2 and D_3 in a 2D domain are constructed by combining the products of these sigmoid gates, thereby localizing the diffusion to specific subdomains of the membrane. For example, the coefficient D_1 is defined as

$$D_1 = S_1\left[\Gamma_{\text{gate}}(x; a_1, f)\,\Gamma_{\text{gate}}(y; y_{R_B}, f) + \Gamma_{\text{gate}}(x; x_{R_C}, f)\,\Gamma_{\text{gate}}(y; d_1, f)\right], \tag{14}$$

where S_1 is a scaling factor, a_1 and d_1 are boundary parameters, and x_{R_C} and y_{R_B} are derived from the centroids of the respective membrane regions. The coefficient D_2 is linearly related to D_1 via

$$D_2 = m\, D_1 + k, \tag{15}$$

with m and k as constants (determined by the geometry of the system), and D_3 is similarly defined as

$$D_3 = S_3 \left[\Gamma_{\text{gate}}(x; e_1, f)\, \Gamma_{\text{gate}}(y; y_{R_F}, f) + \Gamma_{\text{gate}}(x; x_{R_G}, f)\, \Gamma_{\text{gate}}(y; h_1, f)\right], \tag{16}$$

where S_3 is a scaling factor (maximum value of D_3 over the mesh), e_1 and h_1 are boundary parameters, and x_{R_G} and y_{R_F} come from the centroids of the corresponding membrane regions.

The use of sigmoid gate functions as in Eq. (11) provides smooth transitions that mitigate numerical instabilities associated with sharp boundaries. The dynamic adjustment of boundaries, based on the centroid calculation in Eqs. (10), enhances the model's flexibility in representing varying membrane geometries. This methodology is rooted in diffuse-interface and phase-field techniques[2–4] and has been applied in studies of fluid membranes and vesicles.[5] Similar techniques have also been employed to analyze the interaction between membrane shape and intra-surface diffusion.[6]

Entropy Functional Approach to Exponential Convergence

In this section we give a fully rigorous proof that the reaction–diffusion computing unit converges exponentially fast to its unique complex-balanced equilibrium by employing the entropy method of Fellner.[7]

0. Validity of Fellner's hypotheses for the Matrix Computing Unit domain

Since the only requirement for a domain Ω to fulfill the hypotheses is that it should admit a Poincarè constant different from zero, we should prove that this is the case for any possible realization of a Computing Unit. For Poincarè inequality to hold, we require Ω to be at least limited, connected and with Lipschitz border (sufficiently smooth).

Let us recall now the definition of Lipschitz border.

Let $\Omega \subset \mathbb{R}^n$ be an open domain. We say that $\partial\Omega$ is of *Lipschitz class* if, for every point $x^0 \in \partial\Omega$, the following holds:

1. **Cartesian neighborhood.** There exists an open neighborhood $U \subset \mathbb{R}^n$ of x^0.

2. **Local coordinate system.** There is an affine change of variables (rotation plus translation) sending x^0 to the origin and defining new coordinates

$$y = (y', y_n) \ \in \ \mathbb{R}^{n-1} \times \mathbb{R}. \tag{17}$$

3. **Lipschitz function.** There exists a function

$$\phi : B'_r(0) \ \subset \ \mathbb{R}^{n-1} \ \longrightarrow \ \mathbb{R}, \quad B'_r(0) = \{\, y' \in \mathbb{R}^{n-1} : \|y'\| < r\}, \tag{18}$$

which is Lipschitz continuous:

$$\big|\phi(y') - \phi(z')\big| \ \leq \ L\,\|y' - z'\|, \quad \forall\, y', z' \in B'_r(0), \tag{19}$$

for some constant $L > 0$.

In these local coordinates one requires that

$$\begin{aligned} \Omega \cap U &= \{(y', y_n) \in B_r(0) : y_n > \phi(y')\}, \\ \partial\Omega \cap U &= \{(y', y_n) \in B_r(0) : y_n = \phi(y')\}. \end{aligned} \tag{20}$$

That is, in a neighborhood of each boundary point, $\partial\Omega$ is exactly the graph of the Lipschitz function ϕ. This local description rules out arbitrarily sharp cusps or infinite-curvature "corners": every small patch of $\partial\Omega$ has slope bounded by the constant L.

In our case, we can simply define the Ω domain as

$$\Omega = \bigcup_{i}^{I} V_i^{\text{in}} \cup \bigcup_{i}^{J} V_j^{\text{out}} \cup \bigcup_{i,j}^{IJ} V_{i,j} \cup \bigcup_{k}^{2IJ} C_k \tag{21}$$

where all V are non-overlapping regions of fixed finite volume (possibly, as in the main text, squares or cubes) and each C_k is a channel connecting either one inlet to an intermediate compartment or an intermediate to an outlet compartment. In this context we check:

1. **Boundedness.** Each compartment lies in some finite enclosing volume. Since there are only finitely many, their union Ω is contained in a finite rectangle and so is bounded.

2. **Connectedness.** By design every inlet S_i^{in} is adjoined via channels to its intermediates $S_{i,j}^{\text{mid}}$, and each intermediate to its outlet S_j^{out}. Hence any two points in Ω can be joined by a path travelling through each compartment, so Ω is path-connected (and therefore connected).

3. **Lipschitz boundary.** For a 2X2 matrix such as the one in simulated in the main text the boundary $\partial\Omega$ is a finite union of straight-line segments meeting at finitely many nondegenerate angles. Locally near any boundary point one may rotate and translate so that

$$\partial\Omega \cap U \;=\; \{(y', y_n) \in B_r(0) : y_n = \phi(y')\}, \tag{22}$$

where ϕ is piecewise-linear (hence Lipschitz). Equivalently,

$$\big|\phi(y') - \phi(z')\big| \;\le\; L\,\|y' - z'\|, \quad \forall\, y', z' \text{ in a small neighborhood,} \tag{23}$$

with some $L < \infty$. Thus $\partial\Omega$ is globally Lipschitz.

1. Definition of the Entropy Functional

We denote by $\varphi = (\varphi_A, \varphi_B, \varphi_C)$ the concentrations on a bounded Lipschitz domain $\Omega \subset \mathbb{R}^n$, and by $\varphi^\infty = (\varphi_A^\infty, \varphi_B^\infty, \varphi_C^\infty) > 0$ the unique complex-balanced equilibrium determined by

$$\sum_X \int_\Omega \varphi_X \,\mathrm{d}\mathbf{r} = M. \tag{24}$$

For each species $X \in \{A, B, C\}$ define the *relative entropy density*

$$\eta_X(\varphi_X) = \varphi_X \ln \frac{\varphi_X}{\varphi_X^\infty} - \big(\varphi_X - \varphi_X^\infty\big), \tag{25}$$

and the total *relative entropy* (free energy)

$$\mathcal{H}[\varphi] = \sum_{X \in \{A,B,C\}} \int_\Omega \eta_X(\varphi_X)\,\mathrm{d}\mathbf{r}. \tag{26}$$

By construction $\eta_X \ge 0$, with $\eta_X(\varphi_X) = 0 \iff \varphi_X = \varphi_X^\infty$. Moreover $\mathcal{E}$ is a Lyapunov functional for the system.

2. Computation of the Entropy Dissipation

Assume φ_X solves the reaction–diffusion system

$$\partial_t \varphi_X = \nabla\cdot\,(D_X \nabla \varphi_X) + R_X, \qquad \nabla\varphi_X \cdot \nu\big|_{\partial\Omega} = 0. \tag{27}$$

We compute $\frac{d}{dt}\mathcal{E}$ in four steps.

2.1. Time derivative

Since

$$\frac{\mathrm{d}}{\mathrm{d}\varphi_X}\,\eta_X(\varphi_X) = \ln\frac{\varphi_X}{\varphi_X^\infty}, \tag{28}$$

we get

$$\frac{\mathrm{d}}{\mathrm{d}t}\,\mathcal{E} = \sum_X \int_\Omega \ln\frac{\varphi_X}{\varphi_X^\infty}\,\partial_t\varphi_X\,\mathrm{d}\mathbf{r} = \sum_X \int_\Omega \ln\frac{\varphi_X}{\varphi_X^\infty}\big(\nabla\cdot(D_X\nabla\varphi_X) + R_X\big)\,\mathrm{d}\mathbf{r}. \tag{29}$$

2.2. Diffusion part

Integration by parts (using Neumann Boundary Conditions $\partial_\nu\varphi_X = 0$) yields

$$\int_\Omega \ln\frac{\varphi_X}{\varphi_X^\infty}\,\nabla\cdot(D_X\nabla\varphi_X)\,d\mathbf{r} = -\int_\Omega D_X\,\frac{|\nabla\varphi_X|^2}{\varphi_X}\,d\mathbf{r}, \tag{30}$$

so we set

$$\mathcal{D}_{\mathrm{diff}}[\varphi] := \sum_X \int_\Omega D_X\,\frac{|\nabla\varphi_X|^2}{\varphi_X}\,d\mathbf{r}\ \geq 0. \tag{31}$$

2.3. Reaction part

By mass-action and complex balance, for each elementary reaction α, with stoichiometric coefficients ν_X^α one has

$$R_X^\alpha = -\nu_X^\alpha\,(R_f^\alpha - R_b^\alpha), \qquad \sum_X \nu_X^\alpha \ln\frac{\varphi_X}{\varphi_X^\infty} = \ln\frac{R_f^\alpha}{R_b^\alpha}. \tag{32}$$

Hence

$$\sum_X \ln\frac{\varphi_X}{\varphi_X^\infty}\,R_X = -\sum_\alpha\sum_X \ln\frac{\varphi_X}{\varphi_X^\infty}\,R_X^\alpha = -\sum_\alpha\big(R_f^\alpha - R_b^\alpha\big)\,\ln\frac{R_f^\alpha}{R_b^\alpha}. \tag{33}$$

We then define the reaction dissipation as

$$\mathcal{D}_{\mathrm{react}}[\varphi] := \sum_\alpha \int_\Omega \big(R_f^\alpha - R_b^\alpha\big)\,\ln\frac{R_f^\alpha}{R_b^\alpha}\,\mathrm{d}\mathbf{r}\ \geq 0. \tag{34}$$

2.4. Entropy-dissipation law

Collecting diffusion and reaction contributions, (29) becomes

$$\frac{\mathrm{d}}{\mathrm{d}t}\,\mathcal{E} = -\,\mathcal{D}_{\mathrm{diff}}[\varphi] \;-\; \mathcal{D}_{\mathrm{react}}[\varphi] = -\,\mathcal{D}[\varphi], \tag{35}$$

where

$$\mathcal{D}[\varphi] := \mathcal{D}_{\mathrm{diff}}[\varphi] + \mathcal{D}_{\mathrm{react}}[\varphi] \;\geq 0. \tag{36}$$

Thus

$$\frac{d}{dt}\,\mathcal{E}[\varphi(t)] = -\,\mathcal{D}[\varphi(t)]. \tag{37}$$

3. Exponential Relaxation via Fellner's Theorem

We now apply the entropy–entropy-dissipation (EED) framework of Fellner,[8] which, under the following hypotheses, furnishes an explicit EED-inequality:

- *Domain:* Ω bounded, connected Lipschitz.
- *Diffusion:* Uniformly positive coefficients $D_X \geq D_{\min} > 0$.
- *Reactions:* Weakly reversible, complex-balanced network.

Under these conditions there exists $\lambda > 0$, depending only on $D_{\min}$, the Poincaré constant of Ω, and the reaction rates, such that

$$\mathcal{D}[\varphi] \;\geq\; \lambda\,\mathcal{E}[\varphi]. \tag{38}$$

Inserting (38) into the dissipation law yields the differential inequality

$$\frac{\mathrm{d}}{\mathrm{d}t}\,\mathcal{E} \;\leq\; -\,\lambda\,\mathcal{E}, \tag{39}$$

which integrates to

$$\mathcal{E}(t) \;\leq\; \mathcal{E}(0)\, e^{-\lambda t}. \tag{40}$$

Finally, the standard Csiszár–Kullback–Pinsker inequality converts this into

$$\|\varphi(t) - \varphi^{\infty}\|_{L^1(\Omega)} \;\leq\; C_{\mathrm{CKP}}\, e^{-\lambda t}, \tag{41}$$

for an explicit constant $C_{\mathrm{CKP}} > 0$. Thus the computing unit relaxes exponentially fast to its unique equilibrium even through the entropy production functional (9).

Meshing procedures

Meshing is a fundamental step in numerical methods for solving partial differential equations (PDEs), particularly in the Finite Element Methods (FEM) and Finite Volume Methods (FVM). A well-constructed mesh transforms a continuous domain into a discrete representation, allowing computational solvers to approximate solutions with high accuracy. The quality of the mesh directly affects the stability, convergence, and discretization error of the solution.[9,10]

Fine meshes improve accuracy but increase computational cost, while coarse meshes reduce computational demands but may lead to numerical artifacts or inaccurate solutions. Advanced meshing techniques, such as adaptive meshing and error-driven refinement, help optimize computational efficiency by concentrating higher resolution in areas of rapid solution variation. Proper meshing strategies ensure reliable and efficient numerical approximations for PDEs in fluid dynamics, structural mechanics, electromagnetic, and heat transfer simulations. One widely used tool for mesh generation in FEM applications is Gmsh, an open-source software known for its automatic meshing capabilities, adaptive refinement, and compatibility with multiple solvers.[11] We employed gmsh to build the mesh of our simulated system through the usage of the associate graphical user interface (GUI).

The meshing process was carried out using **Gmsh** and followed these steps:

1. A `.geo` file was generated to define the geometry, following the *naïve dimensioning algorithm* proposed by Angerbauer et al.[12]

2. The *mesh* module of the gmsh GUI was used to generate an initial *polygonal (triangular) mesh*, which was subsequently *refined through iterative splittings* to improve resolution.

To achieve an optimal balance between **accuracy** and **computational efficiency**, we found that a **total of 14,819 nodes** was appropriate for our system. However, finer spatial partitions are preferable for generating *high-quality visualizations* of entropy density evolution in video outputs.

References

(1) Kondepudi, D.; Prigogine, I. *Modern thermodynamics: from heat engines to dissipative structures*, second edition ed.; John Wiley & Sons Inc: Chichester, West Sussex, 2015.

(2) Anderson, D. M.; McFadden, G. B.; Wheeler, A. A. Diffuse-interface methods in fluid mechanics. *Annual Review of Fluid Mechanics* **1998**, *30*, 139–165.

(3) Chen, L.-Q. Phase-field models for microstructure evolution. *Annual Review of Materials Research* **2002**, *32*, 113–140.

(4) Cahn, J. W.; Hilliard, J. E. Free energy of a nonuniform system. I. Interfacial free energy. *The Journal of Chemical Physics* **1958**, *28*, 258–267.

(5) Seifert, U. Configurations of fluid membranes and vesicles. *Advances in Physics* **1997**, *46*, 13–137.

(6) Rangamani, P.; Agrawal, A.; Mandadapu, K. K.; Oster, G.; Steigmann, D. J. Interaction between surface shape and intra-surface viscous flow on lipid membranes. *Biomechanics and Modeling in Mechanobiology* **2014**, *13*, 331–344.

(7) Fellner, K.; Prager, W.; Q. Tang, B.; ,Institute of Mathematics and Scientific Computing, University of Graz, Heinrichstrasse 36,8010 Graz, Austria The entropy method for reaction-diffusion systems without detailed balance: First order chemical reaction networks. *Kinetic & Related Models* **2017**, *10*, 1055–1087.

(8) Ref. 7, Thm. 1.2.

(9) Lewis, R. W.; Nithiarasu, P.; Seetharamu, K. N. *Fundamentals of the Finite Element Method for Heat and Fluid Flow*, 1st ed.; Wiley.

(10) Roy, C. Review of Discretization Error Estimators in Scientific Computing. 48th AIAA Aerospace Sciences Meeting Including the New Horizons Forum and Aerospace Exposition.

(11) Geuzaine, C.; Remacle, J.-F. Gmsh: A 3-D finite element mesh generator with built-in pre- and post-processing facilities. *International Journal for Numerical Methods in Engineering* **2009**, *79*, 1309–1331.

(12) Angerbauer, S.; Pankratz, T.; Enzenhofer, F.; Springer, A.; Khanzadeh, R.; Haselmayr, W. Molecular Nano Neural Networks (M3N): In-Body Intelligence for the IoBNT. ICC 2024 - IEEE International Conference on Communications. 2024; pp 4819–4824.
