# Supplementary material for: Thermodynamics Analysis of a Reaction-Diffusion Matrix Multiplication Computing Unit under the Linear Non-Equilibrium Regime
Source: J Phys Chem Lett. 2025 Jun 13;16(25):6293–304. doi: 10.1021/acs.jpclett.5c00834 (PMC12207667; doi:10.1021/acs.jpclett.5c00834)
Supplement: Supplementary file 4 [file jz5c00834_si_004.pdf]

jz-2025-00834z.R1

Name: Peer Review Information for "Thermodynamics Analysis of a Reaction-Diffusion Matrix Multiplication Computing Unit under the Linear Non-Equilibrium Regime"

First Round of Reviewer Comments

Reviewer: 1

Comments to the Author

Referee Report: Thermodynamics of Reaction-Diffusion Matrix Multiplication Units

The manuscript under review presents a compelling thermodynamic framework for understanding matrix multiplication implemented through a chemical reaction-diffusion computing unit. It builds on earlier work in molecular nano-neural networks (M3N) and adds depth by asking whether such systems can operate spontaneously, that is, without continuous external energy supply. This is an important and under-explored question because while many analog computation paradigms have been demonstrated in *silico* or through electrical means, fewer efforts have rigorously interrogated the physical plausibility of such computation within autonomous chemical networks.

One of the key strengths of this work is its focus on entropy production under the linear nonequilibrium regime, framed using tools from local thermodynamic theory. The approach is methodically grounded, employing coupled PDEs and solved numerically to show that convergence to a matrix product output does not require ongoing energy input, provided two thermodynamic constraints are met: the absence of significant cross-diffusion and the presence of steep diffusion gradients (e.g., membranes or functionally similar boundaries). The simulated system performs matrix multiplication by encoding inputs as concentrations of chemical species and observing outputs as steady-state distributions after diffusion and

reaction—a paradigm that may feel abstract but actually aligns surprisingly well with the ethos of reservoir computing.

Indeed, from the perspective of computational neuroscience and machine learning, there's an increasingly accepted view that the brain performs tasks not through precise, clocked operations but via dynamics in high-dimensional, non-linear systems—exactly the kind of behavior that reservoir computing leverages. The classical work of Jaeger (2001) laid the foundation for echo state networks as simple yet powerful models of dynamic computation. The present manuscript touches the edge of this philosophy by showing that chemical substrates can realize similar forms of transformation, perhaps even analogously to how information is processed in biological tissue.

Where the manuscript could be stronger is in explicitly discussing algorithmic scaling. While a 2×2 matrix multiplication is a good starting point, readers will naturally ask whether the approach generalizes to larger matrices or more complex algebraic operations. The authors mention the possibility in passing, but a more explicit analysis—even a heuristic argument about entropy scaling or computational delay with matrix size—would make this a more powerful contribution. In contrast, efforts such as *Memristive Linear Algebra* (Lin et al., arXiv:2407.20539) have demonstrated remarkable scaling of linear operations across large memristive arrays by leveraging the innate physics of Ohm's and Kirchhoff's laws. Comparing this to the current system could help contextualize how well chemical computing fares when viewed through an algorithmic lens, particularly regarding latency, precision, and throughput.

Another relevant point is the connection to natural computation. The design of the computing

unit assumes idealized membranes or reactions, yet nature is full of systems that exploit similar physical principles for organization, signaling, and response—gradients across membranes, reaction fronts, and diffusion-limited aggregation. The authors are right to emphasize that molecular size-based gating and spontaneous relaxation toward equilibrium are real mechanisms in biological systems. This paper moves us a step closer to understanding whether nature itself might "compute" through distributed reaction-diffusion architectures, a hypothesis long considered speculative but here shown to be thermodynamically plausible under specific constraints.

The numerical methods, including FiPy for PDE integration and mesh generation via Gmsh, are appropriate and transparent. The provision of code and simulations is commendable.

Visualizations are clear, though a figure that unifies entropy production across the three regimes would help readers intuitively grasp the effects of membrane sharpness and input distribution on thermodynamic behavior.

In short, this is an ambitious and valuable contribution. It ties together ideas from physical chemistry, theoretical computer science, and neuroscience in a way that opens new avenues of inquiry. With modest additions—especially regarding scaling and context with existing physical computing models—I strongly support publication.

Reviewer: 2

Comments to the Author

The focus of this paper is on the design of chemical computation units, with a focus on matrix multiplication. This problem is important as it forms the basis for the computations required in machine learning; e.g., classification or regression via neural networks. In previous work, the chemical computation framework was developed and shown to work via simulation and macroscale experimental studies. However, existing work did not account for thermodynamic considerations, which are relevant from a physical chemistry perspective.

The focus of the present paper is on the evolution of the entropy over time in the chemical computing system. The authors consider a structure where certain species may diffuse out through a membrane in different compartments which filter by size (a different from the original work in Angerbauer et al.). The resulting reaction-diffusion equation is solved numerically and the entropy (in non-equilibrium regimes) evaluated over time. The authors provide numerical results for different spatial input distributions and sigmoidal diffusion coefficients modeling the membranes.

I have the following comments:

(1) For linear reactions, the (exponential) convergence of linear reaction diffusion equations under non-homogeneous diffusion is now quite well understood for the case with weakly reversible reactions (e.g., with the sigmoidal membrane). I recommend that the authors include discussion of these results; e.g., in

- Fellner, Klemens, Wolfgang Prager, and Bao Q. Tang. "The entropy method for reaction-diffusion systems without detailed balance: first order chemical reaction networks." arXiv preprint arXiv:1504.08221 (2015).

(2) The entropy perspective appears to be helpful to examine the convergence to the equilibrium solution. Although a similar perspective can be obtained from a direct examination of the concentration evolution. The authors mention in the conclusion that the

steepness impacts entropy production, and this appears to be indicated in Fig. 13. However, there is little discussion of this in the numerical results and its implications for the design of the system.

(3) The numerical setup could be clarified. In particular, an explicit description of the physical locations of the compartments and the inflow/outflows would be helpful.

(4) The authors mention that the entropy appears to follow a power-law type relation. Related to point (1), many reaction-diffusion systems have exponential convergence. Can the authors comment of the relationship between these results, and give further implications for the power-law behavior. In particular, is this present for the sigmoidal diffusion model?

Typos:

- above (17) "yealds"

Author's Response to Peer Review Comments:

RESPONSE TO REVIEWERS:

NOTE: All changes to the original main text and supporting material have been highlighted in blue, in order to be immediately visible to reviewers and editor.

REVIEWER 1:

We thank the reviewer for his insightful suggestions, especially regarding complexity scaling and the analogy to Echo and reservoir computing. In response, we have added a dedicated section immediately following the numerical-results block (pp. 28-29) where we:

1. Present heuristic derivations of the hardware complexity, showing that an $I \times J$ multiplier requires $O(IJ)$ compartments and diffusion channels.
2. Derive the constant-time compute latency $\tau_{total} = max\{\tau_{diff,}\tau_{A \to B,}\tau_{B \to C}\}$ under fixed compartment dimensions.

3. Clarify thermodynamic bounds on reaction rates ($k_{fB}, k_{fC} \ll \frac{D}{a^2}$) to maintain uniform concentration profiles.

We have also appended a brief remark (p. 3) to contextualize our approach within classical Echo and reservoir computing architectures, highlighting conceptual similarities and possible avenues for future experimental validation.

These additions should give readers a clearer roadmap for scaling our design and situating it within the broader non-electronic computing landscape.

REVIEWER 2:

The comments of reviewer 2 were extremely important to integrate, in the matrix computing unit analysis, a more rigorous treatment of the asymptotic behaviour. Following the results of Fellner and collaborators, we expanded the final section of our Letter to address all points brought in the reviewing process:

Application of Fellner's Entropy–Entropy-Dissipation Inequality

" For linear reactions, the (exponential) convergence of linear reaction diffusion equations under nonhomogeneous diffusion is now quite well understood for the case with weakly reversible reactions (e.g., with the sigmoidal membrane). I recommend that the authors include discussion of these results; e.g., in - Fellner, Klemens, Wolfgang Prager, and Bao Q. Tang. "The entropy method for reaction-diffusion systems without detailed balance: first order chemical reaction networks." arXiv preprint arXiv:1504.08221 (2015)."

· **Revision:** We have added a dedicated portion of the main text (pp. 23–27), where we recast our reaction–diffusion system into the form of Eq. (19) (p. 23), introduce the quadratic relative-entropy functional (Eq. (21), p. 24), and derive the entropy–dissipation estimate (Eqs. (22)–(24), pp. 24–25). A complete new section of the supporting information file accounts for finer details related to the connection between entropy production and entropy dissipation.

- **Numerical confirmation:** Figure 14 (p. 27) now plots the ratio $\mathcal{D}/\mathcal{E}$ for various sigmoid steepness parameters f and verifies that it always exceeds the theoretical constant Λ_{theory} $\approx 2.3 \times 10^{-7}\ s^{-1}$, computed based on our system parameters.

Role of the Stiffness Parameter in Design Optimization

“ The entropy perspective appears to be helpful to examine the convergence to the equilibrium solution. Although a similar perspective can be obtained from a direct examination of the concentration evolution. The authors mention in the conclusion that the steepness impacts entropy production, and this appears to be indicated in Fig. 13. However, there is little discussion of this in the numerical results and its implications for the design of the system.”

- **Revision:** At the end of the thermodynamics analysis (pp. 19–20), immediately following Figures 12–13, we now explicitly remark that choosing the minimal f that still guarantees convergence is a constrained-optimization problem. For future works we could outline how one could employ a min–max strategy to maximize early-time entropy production subject to fabrication limits on membrane sharpness, but these would out of scope for the present work.

Clarification of Inlet/Outlet Topology

“The numerical setup could be clarified. In particular, an explicit description of the physical locations of the compartments and the inflow/outflows would be helpful.”

- **Revision:** In pp. 13, right before discussing the numerical results, we have expanded the description of the matrix geometry. We now clearly identify inlets and and outlets and refer the reader to Figure 4 . The new text makes unambiguous the A-inflow versus B/C-outflow in the numerical mesh.

Relationship between Power-Law Scaling and Exponential Convergence

“The authors mention that the entropy appears to follow a power-law type relation. Related to point (1), many reaction-diffusion systems have exponential convergence. Can the authors comment of the relationship between these results, and give further implications for the power-law behavior. In particular, is this present for the sigmoidal diffusion model? ”

· **Revision:** In pp. 13–14, following Figure 7, we clarify that the initial power-law regime of $S(t)$ (visible in the log–log inset of Fig. 7) coexists with the long-time exponential relaxation proven by Fellner's theorem. We note that for smooth $D(\boldsymbol{r})$ (Figs. 12–13, pp. 19–23), the simple linear log–log scaling is absent, and while one *could* still fit an effective exponent, it does not yield new physical insight beyond the numerical trends already discussed.

Typographical Corrections

· The typo "yealds" above Eq. (17) has been corrected to "yields" (p. 10).

We hope these revisions fully address the reviewers concerns. We would like to thank them again for the valuable feedback, and we would be honored to receive feedback on the revised manuscript.

Sincerely,
Giuseppe S. Basile and co-authors

jz-2025-00834z.R2

Name: Peer Review Information for "Thermodynamics Analysis of a Reaction-Diffusion Matrix Multiplication Computing Unit under the Linear Non-Equilibrium Regime"

Second Round of Reviewer Comments

Reviewer: 2

Comments to the Author

Many thanks to the authors for their careful revision of the manuscript. I do not have any major recommendations for changes. I would suggest that the authors do another pass for typos; e.g., pg. 21 "one has tho".

Author's Response to Peer Review Comments:

Dear Editor,

thank you for the positive evaluation of our work.

Here is our point-by-point reply:

1. Please submit your publication files without any markups. You may include annotated version(s) of your revised publication file(s) with colored text or highlights indicating the revisions that you have made as "Supporting Information for Review Only."

Reply: Done

2. Reference(s) 19, 22 incomplete. References: In both the main file and the supporting information, fix the style of all references to use JPC Letters formatting (check all references carefully). ***JPC Letters reference formatting requires that journal references should contain: () around numbers; author names; article title (titles entirely in title case or entirely in lower case); abbreviated journal title (italicized); year (bolded); volume (italicized); and pages (first-last). Book references should contain author names; book title (in the same pattern); publisher; city; and year. Websites must include date of access.

Reply: Done

3. Graphics: If a figure has parts labeled (i.e. a, b, etc.), each part must be mentioned in the figure caption.

Reply: Done

4. Author List: Author list must match exactly in three places: (1) manuscript file, (2) supporting information, and (3) ACS Paragon Plus.

Reply: Done

I would suggest that the authors do another pass for typos; e.g., pg. 21 "one has tho".

Reply: Done

Regards

Nunzio Tuccitto
